# The Financial Strength of Our Voluntary Charities

**Published:** 1895-01-19

**Authors:** Henry C. Burdett


### THE INSTITUTIONAL WORKSHOP.

### THE FINANCIAL STRENGTH OF OUR VOLUNTARY CHARITIES.

By HENRY C. BURDETT.

This letter, with the exception of the final paragraph, appeared in the *Times* of the 15th inst.

"The original article in the *Times* and my reply were both concerned with charity *in extremis.* Mr. Rawlings regards the question as if confined to the subject of hospital finance only. The contention of the original article was that the income of charities had decreased, and my reply, as illustrated by figures, showed that the income of all classes of charities was increasing, and that, even after the correction of the transposition in the tables, that of the hospitals had increased by 14½ per cent. My hope and endeavour has always been to increase the income of our hospitals to an adequate extent, and my letter on this occasion was written under the feeling that a note of encouragement at this time must be fruitful in good results to charities. A pessimist view is a hopeless view, and, if allowed to prevail, must in the end destroy the voluntary system. If the present supporters of the voluntary system—*i.e.*, the generous givers of our day and generation—are encouraged, as they must be, by the figures given in my first letter, a feeling of enthusiasm will be aroused which will attract to the cause many new friends who have not as yet learnt the luxury of giving wisely to charity. It follows that a hopeful and true view of the present situation should encourage the hard-worked secretary, because it affords the surest path to the new springs of charity which Mr. Rawlings and his *confrères* naturally desire to discover.

The general experience of charities is that individual contributions are smaller on an average than formerly, although the total income, as I have shown, is larger. Hence, as Dr. Barnardo's table proves, the number of givers must have increased too, and Mr. Rawlings's experience must be an exception.

As to new methods of raising income, the example given by Mr. Rawlings forcibly illustrates the direction in which these fresh fields should be sought for. Localise each big general hospital as much as possible; confine the appeals of special hospitals, like that of which Mr. Rawlings is the secretary, to the people who are particularly interested in their special work; keep a tight hand upon expenditure, and especially upon all items included under the headings "surgery and dispensary"; divide up the lists of old friends and supporters, so as to avoid sending an appeal to any one of them oftener than once in three years; and lastly, treat legacies as income, in periods of depression at any rate, and avoid over building and the undue accumulation of capital. These and many other methods which might be suggested, if pursued with energy under the direction of men of business, must in the end make the metropolitan hospitals as flourishing financially as those in our provincial cities for the most part are. It is true that at the present time there is unfortunately a deficiency of some £25,000, but I trust that there are many willing and wealthy people, who will send special contributions to the metropolitan hospitals during the next few weeks, and so put the balance on the right side. One of the most generous friends to hospitals and charities was the late Mr. Junius S. Morgan, who told me that he never realised the advantage of wealth and the pleasure which it can confer upon the possessor until he learnt to give largely to charitable undertakings of approved value and necessity.

Mr. Rawlings's contention that I am getting a little out of touch with the actual daily work of the hospitals, because I am not now at work within hospital walls, is certainly beside the mark. Having fully mastered the details, and being in constant and increasing communication with the hospital work of all countries, I possess means of observation and judgment which no one actively but solely engaged in a single institution can acquire. Further, an increasing number of people consult me as to the disposal of their gifts to charities, and thus I am enabled to learn the points of view held by the givers. Whilst, therefore, I fully sympathise with the feeling of depression due to the burden of continuous effort imposed upon able and devoted men like Mr. Rawlings, whose services are invaluable to their particular institution, I am able to take a wider view than he can possibly do of the whole situation and of the causes which make for and against increased liberality to hospitals on the part of the public. I know that the pessimistic is not only an inaccurate but a dangerous view. Its undue reiteration has caused a feeling of depression in the minds of most liberal benefactors to hospitals, who were beginning to fear that, do what they will, they cannot supply adequate funds or prevent the hospitals from ultimately lapsing into rate-supported institutions. This view is not justified by the facts, which make for a contrary conclusion, and the sooner it is destroyed, once and for all, the sooner will the income of our hospitals from voluntary sources become adequate to meet their needs and the claims which they undoubtedly have upon the whole community."

There is scarcely a household in the whole country which has not had to reduce its expenditure in recent years, owing to the general fall in the earnings of all classes. My letter showed, however, that the incomes of hospitals had increased by 14½ per cent., whilst the expenditure had increased by nearly three times that amount, although the percentage in the increase of the work done was rather less than more than the percentage in the increase of the expenditure. Mr. Rawlings is perfectly well aware that the present cost of a bed in many London hospitals is surprisingly greater than that of the same class of hospital in other parts of the country. This fact is being taken more and more to heart by the managers of hospitals, and I have reason to believe that the wisest of them have and are endeavouring to curtail expenditure in various directions so as to tide over the present period of depression and scarcity.

